# CHARGE syndrome: genetic aspects and dental challenges, a review and case presentation

**DOI:** 10.1186/s13005-020-00224-4

**Published:** 2020-05-08

**Authors:** Manogari Chetty, Tina Sharon Roberts, Mona Elmubarak, Heidre Bezuidenhout, Liani Smit, Mike Urban

**Affiliations:** 1 Faculty of Dentistry, University of the Western Cape, Private Bag X1, Tygerberg, Cape Town, 7505 South Africa; 2 Division of Molecular Biology and Human Genetics, Faculty of Medicine and Health Sciences, University of Stellenbosch, Stellenbosch, South Africa

**Keywords:** Dental, Genetic, Malformation, Skeletal

## Abstract

**Background:**

CHARGE syndrome (CS) is a rare genetic condition (OMIM #214800). The condition has a variable phenotypic expression. Historically, the diagnosis of CHARGE syndrome was based on the presence of specific clinical criteria. The genetic aetiology of CS has since been elucidated and attributed to pathogenic variation in the *CHD7* gene (OMIM 608892) at chromosome locus 8q12.

**Case presentation:**

A South African female of mixed ancestry heritage, aged 4 years, was referred for dental assessment to the Faculty of Dentistry, University of the Western Cape, in 2018. She had a diagnosis of CHARGE syndrome confirmed by a Medical Geneticist from the Division of Molecular Biology and Human Genetics at the University of Stellenbosch.

The patient had a long prior history of health and developmental problems, with the correct diagnosis becoming apparent over time. She presented with many oral and craniofacial features warranting consideration by the dentist including micrognathia, hypoplastic nasal bones, cranial nerve dysfunction, bruxism, craniofacial anomalies and compromised sensory perception. The treatment was mainly preventive and, although she fed through a percutaneous endoscopic gastrostomy tube (PEG), maintenance of her oral hygiene was necessitated.

**Conclusion:** CS is a multisystem condition and the optimal care for an individual is with a specialist multidisciplinary team. The numerous systemic problems affecting these individuals take precedence in their care, and often there is neglect of their dental concerns. Given the abnormalities frequently present in the oral and craniofacial region, the authors recommend that a team of dental and other medical specialists be involved in the management of individuals with CS.

## Background

The purpose of this article was to review the genetic aspects and dental management challenges of CHARGE syndrome (CS) (OMIM #214800). The history, genetic background, clinical diagnostic criteria of CS are delineated and a case report is presented. The acronym “CHARGE” refers to Coloboma, Heart defect, Atresia choanae, Retarded growth and development, Genital hypoplasia, Ear anomalies/deafness.

Charge syndrome is a rare genetic disorder in which coloboma, choanal atresia or stenosis, cranial nerve dysfunction or anomaly and characteristic ear (external, middle or inner ear) are the major features [1]. Multiple anomalies affecting various organs systems including the cardiovascular and genitourinary systems contribute to the challenging medical management of affected persons [2]. The debilitating general health effects of CHARGE syndrome may be compounded by impairment in cognitive or intellectual functioning, feeding adaptation and behavioural challenges [3].. CHARGE syndrome is a rare genetic condition with an incidence of 1:12,000–15,000 live births [4]. Historically, the diagnosis of CHARGE syndrome was based on the presence of specific clinical criteria [5].

The acronym CHARGE was conferred by Pagon et al.(1981) [6] to describe important clinical features: Coloboma of the eye globe, Heart defects, Atresia of the nasal choanae, Retardation of growth and development, Genitourinary anomalies, and external Ear abnormalities and/or associated hearing loss.

It is now understood that the CHARGE acronym provides an inadequate description of CS. Current clinical criteria rely on the presence of a combination of major and minor features, with the major features being more specific for CS. The most accepted criteria are those described by Blake et al. (1998) [7] and shown in Table 1. Depending on the number of criteria identified, either a definite or a probable/possible diagnosis of CS may be assigned.

**Table 1:** Blake criteria for the diagnosis of CHARGE syndrome

Major criteria	Minor criteria
• Ocular coloboma • Choanal atresia or stenosis • Cranial nerve dysfunction or anomaly • Characteristic ear (external, middle or inner ear)	• Genital hypoplasia • Developmental delay • Cardiovascular malformation • Growth deficiency • Cleft lip and/or palate • Distinctive facial features
**Definitive diagnosis of CHARGE syndrome:** • 4 major features OR • 3 Major and 3 minor features **Probable/possible diagnosis of CHARGE syndrome:** • 1–2 major features and several minor features	

The genetic aetiology of CS has since been elucidated and attributed to pathogenic variation in the *CHD7* gene (OMIM 608892) at chromosome locus 8q12 [7].

The *CHD7* gene is involved in control of gene expression, particularly chromatin remodelling.

Changes in the *CHD7* gene sequence which lead to absent or reduced protein result in disrupted chromatin remodelling, ultimately leading to the multi-organ abnormalities found in CS [8]. *CHD7* seems to be particularly important in controlling the function of neural crest cells, which are pluripotent cells with migratory potential [9]. The neural crest has multiple vertebrate derivatives, including the craniofacial skeleton, the central nervous system (CNS) and associated sensory organs, and parts of the heart. The induction of the neural crest is determined by signalling molecules such as BMP, WNT, FGF and retinoic acid [1, 10, 11]. The process is controlled by a regulatory network of genes including CHD7. Disruption of neural crest development can result in several human disorders known as neurocristopathies including those seen in CS [9].

It has overlapping features with other neurocristopathies such as 22q11.2 deletion syndrome (also known as DiGeorge syndrome or velocardiofacial syndrome) and Kabuki syndrome, which are important differential diagnoses [4]. Use of molecular genetic testing allows less typical cases of CS to be diagnosed and has broadened the phenotypic range of the condition.

The phenotype of CS is variable, with a wide range of possible clinical problems and affected organ systems [10]. Choanal atresia or stenosis causes breathing difficulty that may be life-threatening in the newborn period if both nasal passages are obstructed [12]. Ocular coloboma may cause visual deficit or blindness. Ear or vestibular nerve effects may impact on hearing and/or balance [4]. Other cranial nerve involvement may result in anosmia, abnormal facial expression, and difficulties with feeding and swallowing [13].

There may also be a range of less specific effects. These include congenital heart defects, failure to thrive, delayed development, delayed puberty, tracheoesophageal fistula, kidney abnormalities; disorders of the immune system; scoliosis and limb anomalies may accompany the disorder facial asymmetry and cleft palate [13]. However, not all individuals are affected by intellectual disability, the changes in sight, hearing and/or speech may cause significant learning problems and special needs.

In view of the wide range of organ systems involved, a multidisciplinary approach to management of affected children is necessary [14]. The pattern of congenital malformations and the specific health problems differ amongst affected individuals. For these reasons, we describe the management of a patient with CS, with particular refers to the oral and dental features.

## Case presentation

A South African female of mixed ancestry heritage, aged 4 years, was referred to the Faculty of Dentistry, University of the Western Cape, for dental assessment in 2018. A diagnosis of CHARGE syndrome was confirmed by a Medical Geneticist from the Division of Molecular Biology and Human Genetics at the University of Stellenbosch.

### Diagnostic history

#### Prenatal i

The patient had a lengthy history of health and developmental problems, with the correct diagnosis becoming apparent over time. She was the second child of a healthy non-consanguineous couple. The pregnancy was uneventful and without teratogen exposure. The fetal anatomy scan at 18–22 weeks was missed, but at 36 weeks’ gestation, the sonographic findings included: micrognathia, hypoplastic nasal bone, right renal agenesis and polyhydramnios. Prenatal genetic testing for aneuploidies and 22q11.2 deletion was negative, and a working diagnosis of ‘multiple congenital anomalies’ was proposed.

#### Neonatal course

She was born normally at 37 weeks’, with birth weight of 2980 g (25th centile), length of 45 cm (3-10th) and head circumference of 36 cm (90-97th). In addition to the prenatal features, she had midface hypoplasia and bilateral ear anomalies (protruding ears, absent lobules). She needed mechanical ventilation from birth and remained in the intensive care unit for 5 months. At 3 months, a tracheostomy was performed for upper airway obstruction, resulting from midface hypoplasia. She could neither breastfeed nor bottle-feed, and a PEG was inserted at 4 months.

The girl had limited mouth movements, but an expressionless face. She was unable to suck or swallow, and when oral feeding was attempted, she aspirated feed into her tracheostomy tube. There was minimal drooling. A speech and feeding therapist supervised a period of oral stimulation, but her sucking and swallowing reflexes did not improve. Together, the findings indicated that she had bilateral palsies of cranial nerves 7, 9 and 10. Feeding remained entirely via the PEG, although she occasionally liked to taste food by dabbing it on her tongue. Despite careful attention to diet, her growth decelerated postnatally, and all growth parameters clustered around the 3rd centile.

The eyes were normal externally, but an optic disc coloboma was detected. Audiological assessment indicated that she could localise sound but she required treatment for glue ear with grommets. Despite her prolonged health problems, her motor milestones progressed well - by 9 months of age she had a 2-month deficit.

Her parents described her as a quiet child who had a good relationship with her elder sibling and other children. At examination, she remained unable to talk, though this may have been a reflection of articulation difficulties resulting from cranial nerve palsies and the tracheostomy. She communicated quite well through gestures and understood verbal comments. The patient did not receive any chronic medication but was recalled monthly to evaluate the patency of the PEG. Furthermore, she received antibiotic prophylaxis before any invasive procedure. Currently, she is managed by a multidisciplinary team which includes; occupational therapist, speech/feeding therapist, dietician, ophthalmologist, ENT surgeon, pulmonologist, neurodevelopmental paediatrician,medical geneticist and a dental team.

#### Neonatal investigations and diagnosis

During the neonatal period, a chromosomal microarray showed no significant deletions or duplications of chromosomal material. However, an MRI brain scan showed non-specific changes consistent with ‘brain shrinkage’.

A radiological contrast study at 3 months, indicated that the patient was completely unable to swallow liquids placed in the mouth, however, there was normal peristalsis of contrast material injected into the oesophagus. Additional studies showed that dye in the mouth was aspirated into the trachea, but that there was no evidence of gastro-oesophageal reflux.

Based on clinical and investigation findings, the patient met the clinical criteria for a diagnosis of CHARGE syndrome. The clinical diagnosis was corroborated by molecular investigations: sequencing of the CHD7 gene showed a heterozygous pathogenic variant c.1977delC (p. Lys660Argfs*51).

#### Facial and dental assessment

The main dental complaint was bruxism, and her parents reported that they can hear her grind her teeth at night. Her oral hygiene was poor as her mother wiped her mouth once every morning using a gauze and saline.

**Fig. 1:**
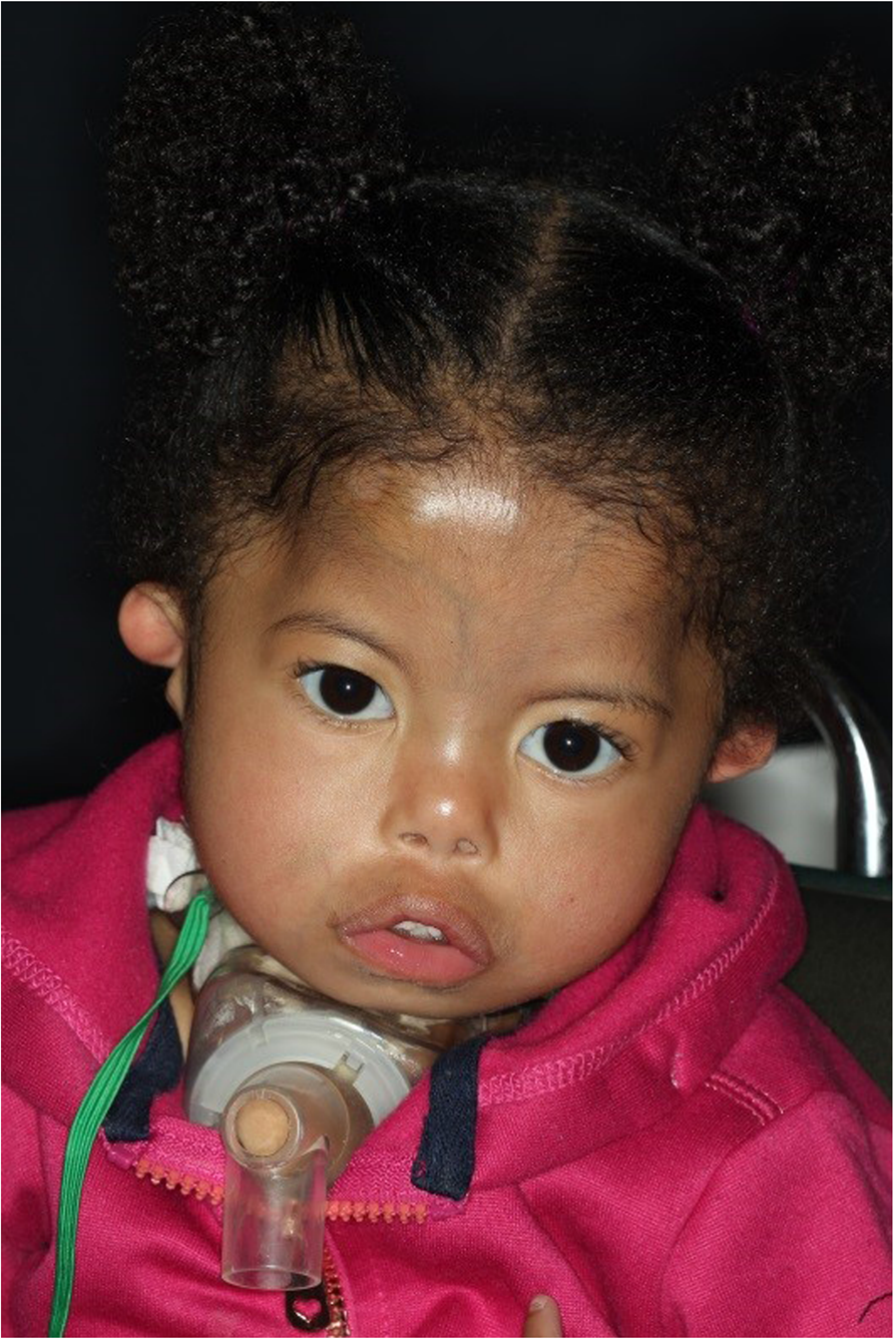
Patient has square shaped face, with mid-facial hypoplasia. Her lips were incompetent with a short philtrum

**Fig. 2:**
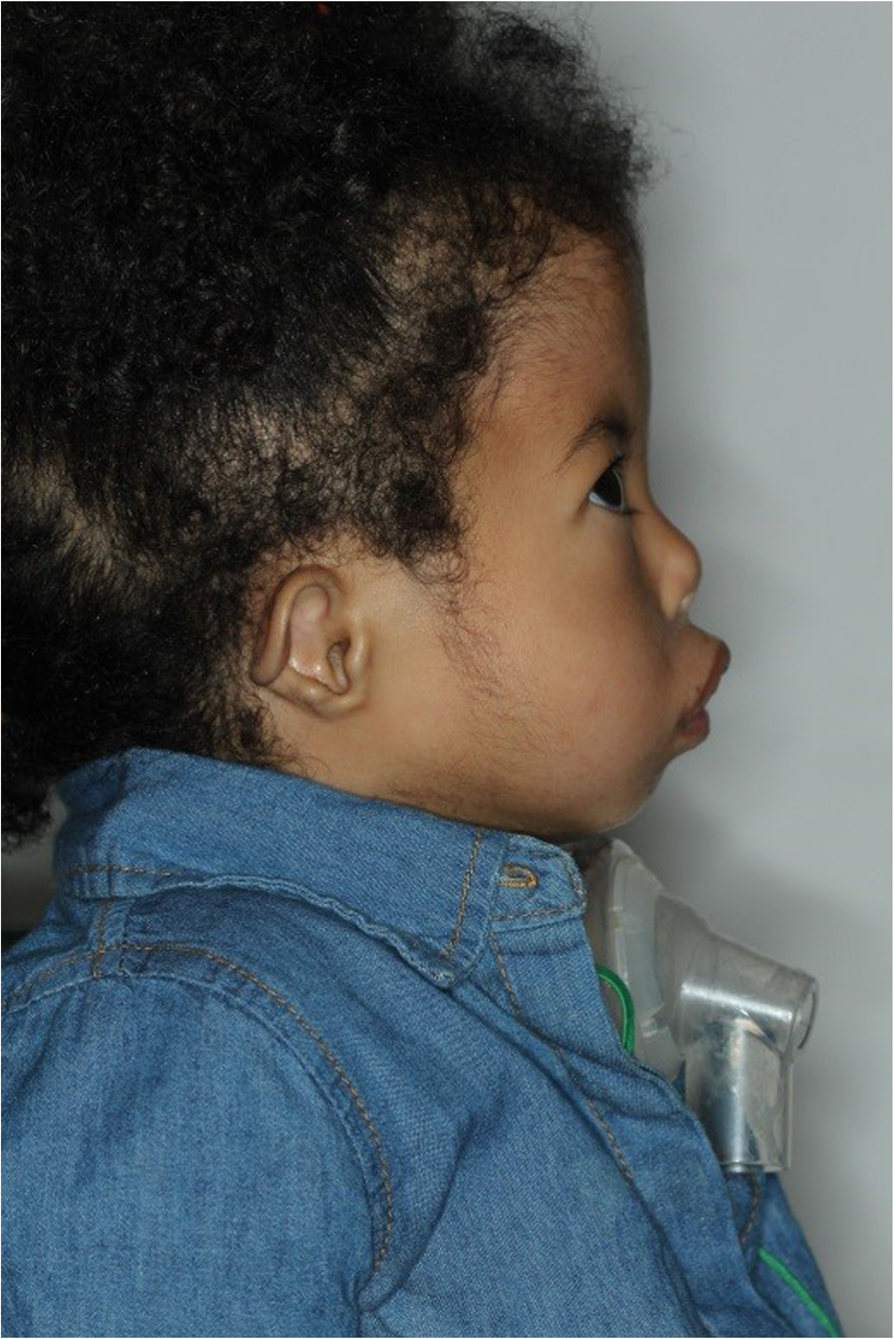
She had marked micrognathia with a tracheostomy device in-situ, and had bilaterally protruding ears that lacked lobules

On examination, the patient has square shaped face, with mid-facial hypoplasia indicated by malar flatness and a small nose with anteverted nostrils. Her lips were incompetent with a short philtrum (Fig. 1). The bilateral lower motor neuron facial nerve palsy resulted in the inability to display any facial expressions. She had marked micrognathia with a tracheostomy device in-situ, and had bilaterally protruding ears which lacked lobules (Fig. 2). The shape of the face and appearance of the ears are typical of CHARGE syndrome.

The child was co-operative and sat in the dental chair without any resistance. She understood instructions but was unable to open her mouth adequately for extensive examination and intra-oral clinical photographs and she did not tolerate certain dental instruments placed in her mouth. It was difficult to gauge the strength of her mouth opening.

**Fig. 3:**
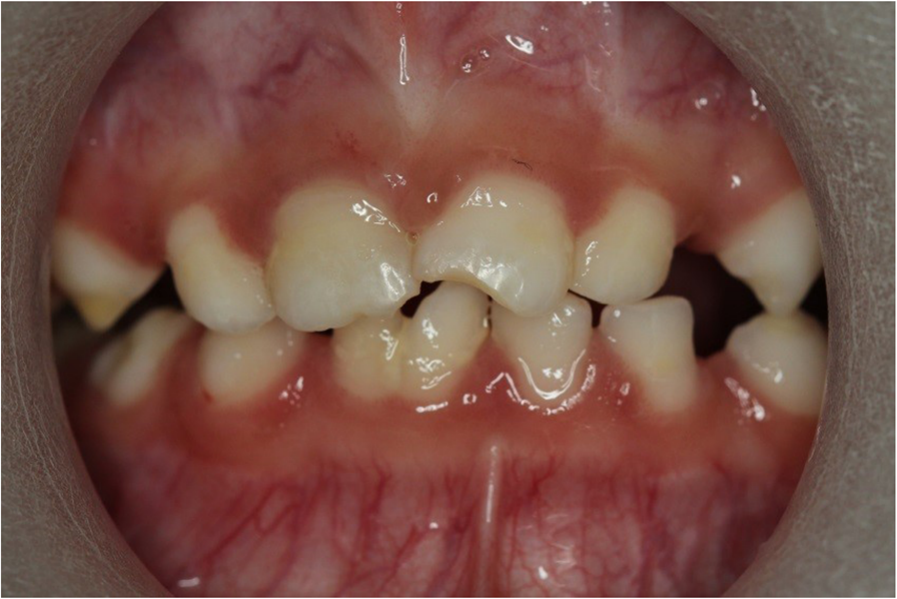
Plaque accumulation and generalized mild to moderate gingival hyperplasia is evident. The incisal edge of her anterior incisor was chipped and there was fusion of her 81 and 82

All primary teeth were present. Although she had poor oral hygiene,visible plaque and generalized mild to moderate gingival hyperplasia, she was caires – free. The incisal edge of her anterior incisor was chipped and there was fusion of her 81 and 82 teeth (Fig. 3). Mild occlusal wear on her posterior teeth was evident, consistent with her bruxing habit. Normal frenal attachment.

#### Dental treatment

The treatment is mainly preventive at this stage and, although she feeds through a PEG tube, her oral hygiene must be maintained to prevent caries and other pathology.

Oral hygiene education was provided to the mother who was advised to brush the child’s teeth twice a day using a soft age-appropriate toothbrush. A simple rubbing technique was demonstrated. A smear layer of toothpaste was advised according to the American Academy of Pediatric Dentistry guidelines [15]. Also, the use of floss if possible between tight contacts in her teeth.

Fluoride was applied on the second visit; an application of 5% sodium fluoride (Clinpro white) varnish using a small brush. The application of fluoride in accordance with the standard protocol of the institution, because of the high caries prevalence in the demographic area. This is usually attributed to poor oral hygiene practices and cariogenic diets of children routinely visiting the dental clinics at the Faculty of Dentistry, UWC. Furthermore, this patient was regarded as a “high risk” candidate for dental caries.

All intraoral examinations using only a mouth mirror were tolerated fleetingly. Ideally, a bite-plate would prevent her bruxism and inhibit excessive forces from being exerted on her temporomandibular joint. However, considering her general health conditions associated to her stage of development, she was unable to tolerate impression material in her mouth and it was decided that if deemed necessary, future dental management will be discussed with a multi-disciplinary dental team after eruption of her permanent teeth. Dental follow-up at 6 monthly intervals was arranged.

## Discussion

Using the Blake criteria (1998) [7], the patient had three major features and three minor features of CS. The major features were ocular coloboma, characteristic external ear, and cranial nerve dysfunction. The minor features were developmental delay, growth retardation and characteristic face. This allowed a clinical diagnosis of ‘definite CHARGE syndrome’.

The clinical diagnosis was later confirmed by genetic testing. A heterozygous variant in the *CHD7* gene was isolated. Since CS results from pathogenic variant in a single copy of *CHD7*, and the *CHD7* gene is located on a non-sex chromosome, the inheritance pattern is autosomal dominant. In this instance, as in most cases of CS, there was no family history. This is because the *CHD7* mutation usually arises de novo in the affected individual, which also means that there is a low recurrence risk in a next pregnancy [16].

Oral health professionals treating children with CS syndrome should be mindful of the several systemic abnormalities associated with the condition as well as other potential challenges, which may influence dental care.

### Dental anomalies found in CS

Dental anomalies seen in CS are those of the shape of teeth includings taurodontism and anomalies of tooth number for example hypodontia and supernumerary teeth. Agenesis, malformations and ectopic eruption of the anterior teeth, in particular, could result in difficultly with phonation and tongue co-ordination. However, the low incidence of this syndrome makes it difficult to delineate a comprehensive dental picture [17]. The dental features reported in the literature were not evident in our patient.

### Challenges in the dental environment

#### Cranial nerve abnormalities

The patient presented many oral and craniofacial features warranting consideration by the dentist. Of particular clinical importance is the involvement of several cranial nerves. The most common cranial nerve abnormalities are those of cranial nerves V, VII, IX, X and XI [13]. In our patient, the trigeminal nerve (V) was not obviously involved. The nerve innervates the muscles of mastication and provides taste to the anterior two thirds of the tongue. Our patient’s ability to taste was preserved and she had limited mouth opening. Her ability to chew could not be assessed, but bruxism may be an indication that the muscles of mastication was strong.

Our patient’s inability to swallow or to protect her airway may have been the result of involvement of the glossopharyngeal (IX) and vagus (X) nerves. The association between CS,feeding difficulties and poor growth is well-documented [4, 13, 16]. Recently it has been documented that cranial nerve dysfunction was the primary clinical feature contributing to feeding difficulties the CS that may result in inadequate sucking, chewing and swallowing and aspiration [18]. Consequently, the presence of these problems in infancy may predict long-term feeding problems that warrants the continuous assistance of a feeding specialist.

Swallowing is a complex, coordinated sequence of activation of over 25 pairs of muscles in the mouth pharynx and oesophagus that is controlled by vagus nerve [19]. The swallowing process protects the airway from aspiration of the food bolus. Defective swallowing in children with CS impede the protection of the airways. For this reason, oral health care workers are cautioned about the possibility of of choking or aspiration of dental material and the risk of anaesthesia-related complications [10]. Blake et al.(2009) [20] assessed 147 anaesthetic events for children with CS and found a 35% incidence of post-operative airways events, 4% of which were for dental procedures. They recommended combining multiple procedures under one anaesthetic wherever possible, as this did not increase the per-procedure risk of post-operative complications.

#### Bruxism

Bruxism is known to occur in CS. Inchingolo et al. (2014) [17] reported constant nighttime bruxism in at least two of seven cases. Young children self-stimulate in order to learn about their bodies and the environment. This need is more intense in children with multisensory impairment, a fact which may be relevant to the development of bruxism in CS [21].

#### Sensory perception

The effects of CS on organs of sensory perception and communication are important for a dental practitioner. Problems with sensory receptors of the eye, the ear, and frequently also the vestibular apparatus may make functioning for the affected individuals very challenging, especially where severe combined deficits of balance, hearing and vision impact on motor skills and communication [21]. Within the realms of dentistry, appropriate therapy would necessitate close collaboration with ‘deafblind’ specialists [22].

In our patient balance was normal, and vision and hearing not severely affected. However, communication was hampered by lack of facial expression and lack of speech, both expected to be permanent. Although plastic surgery techniques may cosmetically improve facial expression, this may not be crucial since our patient does not drool and effectively uses gestures to convey emotion. While 75% of patients with CS have intellectual disability, in this patient the lack of speech may relate to articulation rather than intellectual difficulties. Assessment by a neurodevelopmental paediatrician is essential, both to determine intellectual potential and to consider the role of alternative communication methods, for example using specific sign language or assisted communication devices.

#### Cognitive function

If there is impaired cognitive function, the effects on behaviour need to be considered. There may be very goal directed and persistent behaviour, which often increases under stress. In addition, self-regulation may be poor, with easy loss of behavioural control and difficulty shifting attention and moving onto new things. The anticipation of pain may lead to elevated levels of anxiety which can result in lowered tolerance to pain [23]. These factors need to be taken into account during dental examination and therapies. It may require therapies to be adjusted and guide dental surveillance on potential future dental needs.

#### Craniofacial anomalies

A number of other oral and dental features of CS have been previously described [17]. There is a short upper lip, with lip incompetence and oral breathing. In a retrospective study between 1998 and 2016, Isaac et al. (2017) [24] reported that 11 of 44 patients with CS presented with a cleft of either the lip, palate or both. Infants with CS and clefts is at an increased risk for feeding difficulty and speech. Furthermore, breathing may be obstructed by unilateral or bilateral choanal atresia.

Cranio-cervical junction abnormalities are common in CS [24]. These are demonstrated by a high prevalence of basioccipital hypoplasia and basilar invagination. Careful manipulation of the head during dental management is necessary in order to prevent possible life-threatening consequences of basilar invagination.

#### Allergies

Kong and Martin (2018) [25] a high percentage of allergies in individuals with CS (approximately 48%). The allergens included food and drugs and manifested in contact allergies, rhinitis and asthma. In dental context, the use of gloves and dental materials may initiate atopic reactions in patients with CS and cautioned is required when treating them.

## Conclusion

The dental and craniofacial anomalies often present in CS result in breathing and swallowing difficulties, periodontal disease due to poor oral hygiene and the traumatic stimulation of bruxism, and the lack of co-ordination and paralysis of the facial musculature. Dentofacial orthopaedics maybe delayed in patients who are medically unstable.

CS is a multisystem condition and the optimal care for an individual is with a specialist multidisciplinary team. The multiple systemic problems affecting these individuals take precedence in their care, and often there is neglect of their dental concerns. Given the abnormalities frequently present in the oral and craniofacial region, the authors recommend that a team of dental and other medical specialists be involved in the management of individuals with CS. The dental practitioner, as part of this multidisciplinary team, can add value to the care and quality of life of individuals with CS, but must also mindful of the potential risks associated with such care, for example related to anaesthesia. This case report contributes to the limited published information on the dental concerns in this genetic condition and highlights the need for further research.

## Data Availability

All supporting data will be made available on request by the Editor-in-chief.
